# Molecular Profiling and Clinical Outcome of High-Grade Serous Ovarian Cancer Presenting with Low- versus High-Volume Ascites

**DOI:** 10.1155/2014/367103

**Published:** 2014-05-25

**Authors:** Tomer Feigenberg, Blaise Clarke, Carl Virtanen, Anna Plotkin, Michelle Letarte, Barry Rosen, Marcus Q. Bernardini, Alexandra Kollara, Theodore J. Brown, K. Joan Murphy

**Affiliations:** ^1^Division of Gynaecologic Oncology, Department of Obstetrics and Gynaecology, University of Toronto, Toronto, ON, Canada M5G 2M9; ^2^Department of Obstetrics and Gynaecology, Trillium Health Partners, 2200 Eglinton Avenue West, Mississauga, ON, Canada L5M 2N1; ^3^Department of Laboratory Medicine and Pathology, University of Toronto, Toronto, ON, Canada M5G 2M9; ^4^Department of Laboratory Medicine and Pathology, University Health Network, Toronto, ON, Canada M5T 2M9; ^5^Princess Margaret Genomics Centre, University Health Network, Toronto, ON, Canada M5G 1L7; ^6^Department of Laboratory Medicine and Pathology, Trillium Health Partners, Mississauga, ON, Canada L5M 2N1; ^7^Hospital for Sick Children and Department of Immunology, University of Toronto, Toronto, ON, Canada M5G 1X8; ^8^Division of Gynaecologic Oncology, Department of Obstetrics and Gynaecology, Princess Margaret Cancer Centre, Toronto, ON, Canada M5G 2M9; ^9^Lunenfeld-Tanenbaum Research Institute, Mt. Sinai Hospital, Toronto, ON, Canada M5T 3L9

## Abstract

Epithelial ovarian cancer consists of multiple histotypes differing in etiology and clinical course. The most prevalent histotype is high-grade serous ovarian cancer (HGSOC), which often presents at an advanced stage frequently accompanied with high-volume ascites. While some studies suggest that ascites is associated with poor clinical outcome, most reports have not differentiated between histological subtypes or tumor grade. We compared genome-wide gene expression profiles from a discovery cohort of ten patients diagnosed with stages III-IV HGSOC with high-volume ascites and nine patients with low-volume ascites. An upregulation of immune response genes was detected in tumors from patients presenting with low-volume ascites relative to those with high-volume ascites. Immunohistochemical studies performed on tissue microarrays confirmed higher expression of proteins encoded by immune response genes and increased tumorinfiltrating cells in tumors associated with low-volume ascites. Comparison of 149 advanced-stage HGSOC cases with differential ascites volume at time of primary surgery indicated low-volume ascites correlated with better surgical outcome and longer overall survival. These findings suggest that advanced stage HGSOC presenting with low-volume ascites reflects a unique subgroup of HGSOC, which is associated with upregulation of immune related genes, more abundant tumor infiltrating cells and better clinical outcomes.

## 1. Introduction

Epithelial ovarian cancer (EOC) is the leading cause of gynecologic cancer-related death in developed countries, with nearly a quarter million women diagnosed worldwide annually [1]. Of the various EOC histotypes, which have distinct precursor lesions, genomic profiles, and clinical course [2, 3], high-grade serous ovarian cancer (HGSOC) accounts for the majority of cases and a disproportionate number of deaths. Adding to the complexity, recent large-scale gene expression studies identified at least four molecular subtypes within HGSOC [4, 5], with some evidence associating these subtypes with differences in overall patient survival [4, 6].

Ovarian cancer is typically diagnosed at an advanced stage, with high-volume ascites a common presenting feature [7]. While some studies have indicated better prognosis in cases presenting with no ascites, most reports to date have grouped all histological subtypes and tumor grades together. Such an approach makes it difficult to assess if differences in ascites volume are an independent predictor of survival and better surgical outcome. The association of ascites with poor outcome in EOC could reflect the fact that HGSOC is an aggressive cancer that tends to present with high-volume ascites and carries poor prognosis compared to other histological subtypes and low-grade disease [8–11]. For this reason, we focused on a homogeneous group of patients diagnosed exclusively with HGSOC, to assess the significance of differences in ascites volume at the time of diagnosis. Although ascites often resolves early in therapy, reaccumulation occurs frequently and becomes a significant quality of life issue, particularly with chemoresistance and disease progression. Shortness of breath, abdominal bloating, pain, nausea, and early satiety caused by ascites contribute to cachexia with eventual compromise of the patient's mobility and often with respiratory distress and bowel obstruction [12]. While the pathogenesis of malignant ascites is incompletely understood, increased vascular permeability and tumor neovascularization due to high concentrations of vascular endothelial growth factor (VEGF) and decreased rates of lymphatic drainage are considered critical [13–17]. Despite its clinical importance, ascites volume has not been captured as a parameter in molecular profiling studies.

In the present study, we focused on the impact of differences in ascites volume on patients diagnosed with advanced stage HGSOC. We compared gene expression profiles in a discovery cohort of these tumors. Our analysis revealed a unique subset of immune-related genes upregulated in the low-volume ascites group. Immunohistochemistry performed on a larger cohort of primary tumors validated these results and showed increased number of tumor infiltrating immune cells in the low-volume ascites group. This group also had better surgical outcome, defined as optimal (<1.0 cm residual tumor) versus suboptimal cytoreduction, and overall survival that was consistent with a stronger tumor immune response seen in those patients.

## 2. Material and Methods

### 2.1. Whole Genome Transcriptome Profiling

A discovery cohort of snap-frozen, stage IIIC primary HGSOC specimens from 12 patients presenting with low- (≤200 cc) or high-volume (≥1000 cc) ascites was obtained from the University Health Network Biobank. A gynecology pathologist (BC) reviewed each specimen to confirm the diagnosis and ensure presence of more than 70% epithelial tumor cells. The University Health Network Research Ethics Board approved this study and all patients consented to the use of their tissue and clinical data for research.

RNA was extracted from tumor tissue using an RNeasy Mini Kit (Qiagen). Quality and quantity of RNA as well as cDNA were confirmed prior to hybridization to Illumina HumanHT-12 v4r2 BeadChip microarrays. Only samples passing quality control metrics in the Illumina BeadStudio and R (version 2.14.1; Lumi Bioconductor package) software programs were included in the final analysis (9 of the 12 low-volume and 10 of the 12 high-volume ascites). Array data were converted to logs, quantile, and median-normalized and analyzed for differential expression between groups using GeneSpring (v12.1, Agilent). Unsupervised hierarchical clustering using average linkage rules and a Pearson centered distance metric was performed to assess the overall degree of gene expression similarity among samples [18]. All probes were filtered prior to analysis to remove those showing little or no signal in either sample group. Only probes reacting with at least 80% of samples in either group, with expression in the 20–100th percentile of measured signal values, were retained. A moderated student's* t*-test [19] without multiple testing corrections was used to identify probes whose mean expression was different between low- and high-volume ascites samples. A Westfall and Young Family Wise Error Rate (FWER) multiple testing correction was also applied to the moderated* t*-test. All probes found significant were ranked by fold change. Gene ontology (GO) analysis using a hypergeometric test with a false discovery rate (FDR) cutoff of *q* < 0.2 was used to find significantly altered categories. For gene set enrichment analysis (GSEA), version 3.1 of mSigDB was used with a cutoff FDR of *q* < 0.1 [20] using all unfiltered probes on the array. Gene expression array data have been deposited in the gene expression omnibus repository, accession number GSE51831.

### 2.2. Validation by Immunohistochemistry

A tissue microarray was constructed using a semiautomated TMArrayer (Pathology Devices, Inc.) from archived formalin-fixed paraffin-embedded tumors from a total of 54 patients with stages III-IV HGSOC presenting with low- or high-volume ascites, including the 24 patients initially considered for the discovery cohort plus an additional 30 patients identified in the ovarian cancer database at Princess Margaret Cancer Center, Toronto, ON. Only patients with sufficient archived primary and metastatic tissue were included. Two cores of each tissue were selected. Three cores were excluded prior to analysis due to poor quality; hence a total of 51 cores were included in the final analysis. Immunohistochemical studies were performed for detection of proteins encoded by genes highly expressed in the low-volume ascites group. Sections (4 *μ*m thick) of the tissue microarray were deparaffinized and antigen retrieval or unmasking procedures were applied, if necessary, by heating the sections in 10 mM citrate buffer at pH 6.0 or Tris-EDTA buffer at pH 9.0 using a microwavable pressure cooker. Endogenous peroxidase was blocked with 3% hydrogen peroxide. After blocking with appropriate serum, sections were incubated with primary antibody using previously optimized conditions. Primary antibodies included anti-CD3 (1 : 500, cat. A0452, DAKO), anti-CD8 (1 : 200, cat. NCLCD8-4B11, Vector Labs), anti-CD20 (1 : 300, cat. M0755, DAKO), anti-CD74 (1 : 500, cat. Ab9514, Abcam), anti-CD48 (1 : 7500, cat. 962-M01, Novus Biological), anti-TAP2 (1 : 1000, cat. HPA001312, ATLAS), and anti-HLA-DR (1 : 7500, cat. NB120-17844, Novus Biological). A Super Sensitive Polymer-HRP Kit (BioGenex QD440-XAK) was used to amplify primary antibody staining. Immunostaining was visualized using freshly prepared diaminobenzidine solution (DAKO). Sections were lightly counterstained with Mayer's hematoxylin.

Tissue cores stained for HLA-DR, CD74, CD48, and TAP2 were scored as zero (no staining of tumor), one (intermediate staining), or two (strong staining in ≥50% of tumor) by a pathologist blinded to patient clinical information. The tissue microarray was also stained for markers of infiltrating T (CD3, CD8) and B cells (CD20). For each patient, one area of primary tumor and one area of metastasis were selected after scanning all cores for tumor epithelium mostly enriched with tumor infiltrating leukocytes (TILs), in accordance with a previously described protocol [21]. The number of TILs was counted in one representative high power field up to a maximum of 50 TILs per high power field. Staining intensity scores for tumor antigens were described using frequencies and proportions and compared between patients with low- and high-volume ascites using the Wilcoxon rank-sum test and between primary tumor and metastases using the Wilcoxon signed-rank test, with each sample acting as its own control. Immune cell scores were compared between the two patient groups using Wilcoxon rank-sum test. Generalized estimating equations were used to determine ordinal and logistic regression parameters while adjusting for repeated measurements on patients to compare those with low- and high-volume ascites on pooled tumor staining scores and on pooled numbers of immune cells in the epithelium. Statistical significance was set to *P* < 0.05. All analyses were implemented using SAS software, version 9.3 TS level 1 M1.

### 2.3. Patient Outcome Assessment

A search of the Princess Margaret Cancer Center Ovarian Cancer Database identified 240 stages III-IV HGSOC cases that underwent up-front cytoreductive surgery between January 2003 and August 2011. Clinical data extracted from real-time synoptic operative reports and/or electronic medical records for these patients included age at diagnosis, FIGO stage, ascites volume at time of surgery, surgical outcome, and date of death. Where applicable, date of death or survival duration as of April 2012 was validated using the Ontario Cancer Registry. Only those patients with a clear indication of ascites volume ≥1000 cc (high-volume) or ≤200 cc (low-volume) were included in further analysis (*n* = 149). Continuous variables were compared between patients with high- and low-volume ascites using the Wilcoxon rank-sum test. Categorical variables were compared using Fisher's exact test. Overall survival was measured from the date of surgery to the date of death from any cause. Patients alive at last followup were censored. The log-rank test was used to compare outcomes for patients with high- and low-volume ascites, and a Cox proportional hazards regression model was used to compare the two groups while adjusting for stage. Kaplan-Meier plots were generated to estimate one-, three-, and five-year survival probabilities. Statistical significance was set to *P* < 0.05.

## 3. Results

### 3.1. Gene Expression Profiling Reveals a Distinct Signature for Tumors Associated with Low- versus High-Volume Ascites

**Figure 1:**
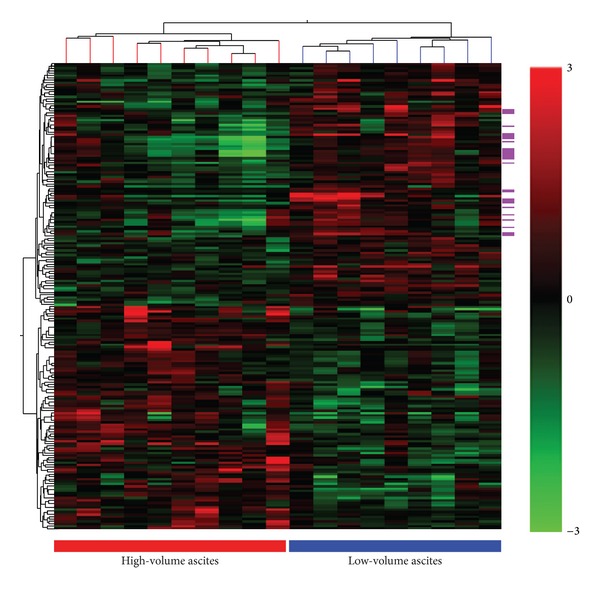
Gene expression profiling of a discovery cohort reveals an immune gene signature for HGSOC tumors presenting with low-volume ascites. Hierarchical clustering of patient samples based on 198 probes differentially expressed by ≥1.5-fold as determined by a moderated* t*-test (*P* < 0.05) in tumors associated with high- and low-volume ascites. Each line of the cluster tree shown at the top represents one patient sample. The ascites volume group is indicated by the bar at the bottom and by the line color. Magenta bars on the side indicate probes corresponding to the 20 genes that overlap with genes within the TCGA immunoreactive group and are upregulated in the low-volume ascites group.

Nine samples with low-volume ascites and ten samples with high-volume ascites met the requirements for RNA and cDNA quality and constituted the discovery cohort. Median age for patients with low- and high-volume ascites was 68 (range 44–84) and 58.5 years (range 46–85), respectively (*P* = 0.57). All patients were diagnosed with stage IIIC disease. Unsupervised two-way hierarchical cluster analysis of both the entire set of the filtered array probes (35433 probes) and a subset of probes (371 probes) that showed the most overall variability (overall standard deviation >1.0) did not segregate the samples according to ascites volume (data not shown). An uncorrected moderated* t*-test found 198 probes statistically different between ascites volume groups using *P* < 0.05 and a minimum fold change of 1.5. Of these, 103 probes representing 98 unique genes were upregulated in the low-volume ascites tumors and 95 probes representing 84 unique genes were upregulated in the high-volume ascites tumors (see Supplemental Table S1 in Supplementary Material available online at http://dx.doi.org/10.1155/2014/367103). A clustering of samples based upon these 198 probes is shown in Figure 1. Fifteen probes using a Westfall and Young FWER corrected moderated* t*-test were found to be significant using a *P* < 0.1 threshold and a minimum fold change of 1.5. These overlapped entirely with the 198 probes found with the uncorrected moderated* t*-test (Supplemental Table S1).

Using GO analysis, an enrichment of GO terms such as antigen processing and presentation, MHC protein complex (particularly MHC II), and cytokine activity was found in the low-volume ascites group (Table 1). Not surprisingly, the 9 (out of 15) probes found upregulated in the low-volume ascites using the Westfall and Young corrected results were also enriched for immune related categories (data not shown). This enrichment of immune-related categories in the low-volume ascites group was further confirmed with GSEA testing (Supplemental Data Table S2), which uses* a priori* defined gene sets to find statistically significant (FDR-corrected) changes between two defined groups of samples. In high-volume ascites cases, GO analysis revealed genes associated with extracellular matrix (Supplemental Table S3).

### 3.2. Increased Immune Response in Tumors from HGSOC Patients with Low-Volume Ascites

**Figure 2:**
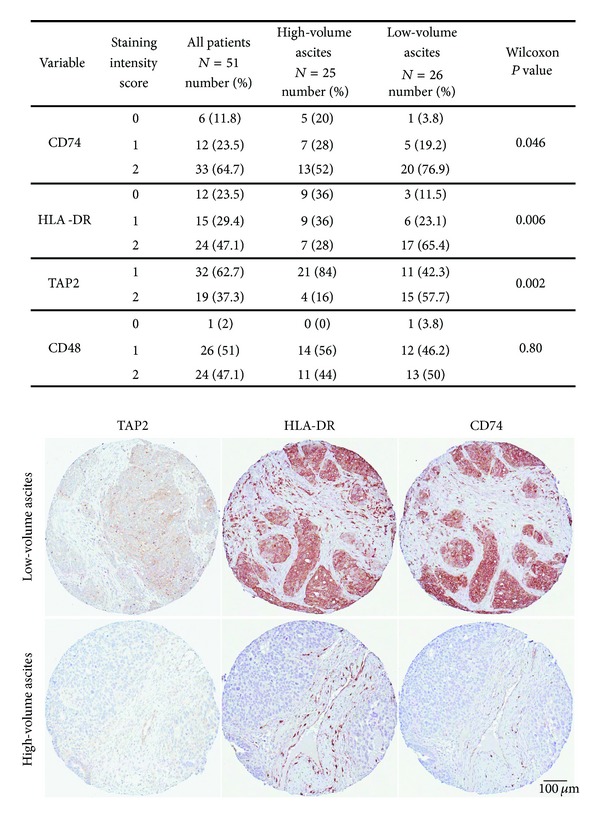
Epithelial scoring for CD74, HLA-DR, TAP-2, and CD48 expression and immunohistochemical staining in representative cases of low- and high-volume ascites associated primary tumor samples. Immunohistochemistry was performed on tissue microarrays containing a total of 54 tumors associated with high- and low-volume ascites. Magnification 9x.

CD74, HLA-DR, and TAP2 were expressed at significantly higher levels in the epithelium of cancer cells derived from tumors of patients with low- versus high-volume ascites (Figure 2; *P* = 0.046, *P* = 0.006, and *P* = 0.002, resp.), consistent with our microarray data. CD48 staining did not show the differential expression suggested by the RNA data. Staining intensity for the four antibodies was similar between the primary tumors and the corresponding metastatic lesions. While tumor infiltrating T lymphocytes were more abundant than B cells in the tumor epithelium, infiltrating T and B cells were both more common in the tumor epithelium of the low-volume ascites group based on staining for CD20, CD8, and CD3 (*P* = 0.02, *P* = 0.001, and *P* = 0.01) (Table 2). The number of TILs did not differ between primary tumors and corresponding metastatic lesions.

### 3.3. Low-Volume Ascites Is Associated with Better Surgical Outcome and Survival

**Figure 3:**
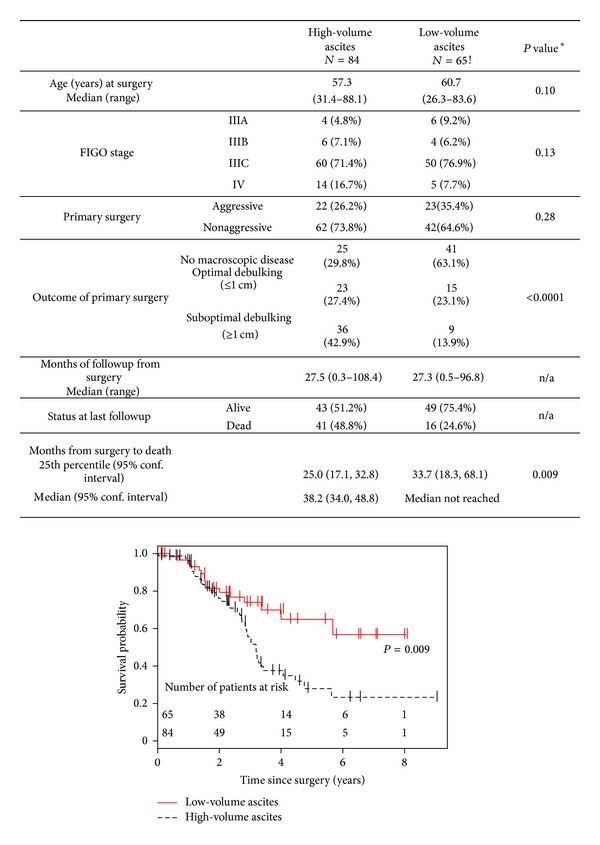
Comparison of clinical parameters of patients included in outcome analysis. *P* values shown in the table were determined by Fisher's exact test for categorical variables, the Wilcoxon rank-sum test continuous and ordered variables, and the long-rank test for days to death. n/a: statistical test not applied. The predicted probability of overall survival in patients with low- and high-volume ascites diagnosed with stages III-IV HGSOC is shown by the Kaplan-Meier plot. Data included in this plot were analyzed by a log-rank test.

Overall 149 patients were included in the clinical data and outcome study: 65 with low-volume ascites and 84 with high-volume ascites. Mean age at diagnosis was similar and over 70% of patients were stage IIIc at the time of diagnosis in the two groups (Figure 3). While a higher percentage of patients with low- as compared to high-volume ascites had undergone aggressive surgery (defined as at least one of the following: diaphragmatic stripping, peritoneal resection, bowel resection, or splenectomy) to achieve optimal surgical outcome, this difference was not statistically significant (73.8% versus 64.6%; *P* = 0.28). However, the clinical course of disease differed between the two groups; the outcome of primary surgery was better for the low-volume ascites group with 63.1% of patients having no macroscopic evidence of disease at the end of surgery, compared with only 29.8% in the high-volume ascites group. Overall, only 13.9% of the low-volume ascites cases, as compared to 49.2% of the high-volume ascites cases, were suboptimally debulked (*P* < 0.0001). This difference in debulking success remained after adjusting for stage. Moreover, 75.4% of patients in the low-volume ascites group were alive at last followup (median = 27.3 months) versus 51.2% in the high-volume ascites group (median = 27.5 months). Since the 50th percentile survival had not yet been reached in the low-volume ascites group, we compared the 25th percentile for overall survival, which showed a value of 33.7 months versus 25 months for the low- versus high-volume ascites groups (*P* = 0.009) (Figure 3). After adjusting for stage, the high-volume ascites group was still associated with higher risk of death (hazard ratio = 2.1, 95% confidence interval: 1.18, 3.78, *P* = 0.01) (Figure 3).

## 4. Discussion

Our results demonstrate molecular differences between HGSOC associated with low-volume ascites compared to those with high-volume ascites. The most significant finding is an upregulation of immune response genes in the low-volume ascites group. The corresponding proteins are involved in immune response: HLA-DR, HLA-DM, and CD74, which play a crucial role in HLA class II antigen processing and presentation; HLA-A and HLA-F, MHC class I molecules, and their intracellular transport proteins TAP1 and TAP2, involved in stimulation of cytotoxic responses; chemokines such as CXCL9, CXCL10, and CXCL16 and cytokines such as CCL5, which participate in T-cell activation and chemotaxis. Consistent with the microarray transcript data, immunohistochemical staining confirmed increased expression of HLA-DR, CD74, and TAP2 proteins in the tumor epithelium of the low-volume ascites group. In support of these differences in immune response gene expression, our results demonstrate that low-volume ascites tumors are characterized by more abundant infiltrating immune cells. In addition, our data show that patients presenting with HGSOC and low-volume ascites are more likely to have successful cytoreductive surgery and to experience longer survival than those presenting with high-volume ascites, despite similar stage and grade at presentation.

It has long been established that EOC can stimulate host immune response and that the presence of infiltrating T cells, particularly CD8^+^ cytotoxic T lymphocytes (CTL), is associated with a better outcome [22–26]. In accordance with these studies, our results indicate better clinical outcome for the low-volume ascites group, whose tumors are characterized by more abundant tumor infiltrating cells. High expression of HLA-DM and HLA-DR by the tumor epithelium of HGSOC correlates with tumor infiltrating T cells and better overall survival [27]. HLA class I antigens bind tumor-associated peptides during their intracellular assembly, which are then presented to CTL. In general, EOC lesions display downregulated expression of HLA class I or the processing intermediates, TAP1 and TAP2. In view of the crucial role of the HLA class I complex in presenting tumor-associated peptides to CTL, it is not surprising that downregulation of HLA class I and its processing machinery is associated with advanced stage and disease progression [28] and has a negative impact on patient survival [29]. Thus, our finding that both HLA classes I and II genes are upregulated in tumors from patients with low- versus high-volume ascites could help to explain their better clinical outcome. This interpretation is supported by a recent cross-platform study of six gene array datasets (including the TCGA and the Tothill datasets) by Yoshihara et al. [6] that found decreased overall survival in HGSOC associated with reduced expression of immunoreactive genes in the tumor.

Large-scale, genome-wide gene expression profiling studies of HGSOC by Tothill et al. [4] and TCGA [5] indicate the existence of distinct molecular subgroups, with both studies identifying a subgroup enriched in immune response genes. While these studies incorporated a wide range of presenting clinical parameters, ascites volume was not included. A preliminary comparison of our upregulated genes in tumors associated with low-volume ascites with the genes upregulated in the TCGA immune group indicates an overlap (shown in Figure 1) that is not expected by chance alone (*P* < 0.01, hypergeometric test). The number of samples available for our discovery cohort was limited in size due to the variable capturing and inconsistent quantification of ascites volume in tumor banks and clinical databases, which resulted in our inability to find an external dataset to validate our findings. Nonetheless, our results are highly suggestive that low-volume ascites may be an associating clinical parameter of the “immune” molecular subgroup of HGSC.

The surgical outcome at the end of primary cytoreductive surgery is one of the most important independent predictors of survival for advanced stage EOC. While resection of all macroscopic residual tumor is the optimal goal, debulking of tumor lesions to <1.0 cm in their greatest dimension is considered beneficial [30]. Other independent prognostic factors identified by a large retrospective study of 1895 patients diagnosed with stage III EOC include age, histological type, and performance status [31]. In our study, age was similar between the two groups and histological type was restricted to HGSOC; performance status was not addressed. Our results show that patients with advanced stage HGSOC presenting with low-volume ascites have improved survival compared to patients with high-volume ascites. It remains unclear as to whether this is the result of improved surgical outcome, an enhanced immunoresponsiveness, or an interaction between these two factors.

## 5. Conclusion

This study shows that HGSOC presenting with low-volume ascites has molecular features distinct from HGSOC presenting with high-volume ascites and is characterized by an enhanced immunoreactive phenotype, better surgical outcome, and prolonged overall survival. While further prospective studies are required, our findings suggest that these patients are likely to achieve favorable outcome at the end of primary cytoreductive surgery. Our results also indicate that adjuvant immunotherapy may be a reasonable future approach for the treatment of ascites. We believe that ascites volume is an important clinical parameter that should be accurately captured to enable future research on differences in ascites volume in advanced ovarian cancer.

## Supplementary Material

Three supplemental tables are available online. The first of these provides a complete listing of the genes found differentially expressed between HGSOC tumors associated with low-volume ascites and tumors associated with high-volume ascites. The second table presents results of an independent gene set enrichment analysis (GSEA) of the gene expression data, which further supports the interpretation that genes up-regulated in tumors associated with low-volume ascites are involved in immune response. The third Table presents the results of a gene ontology (GO) analysis of genes upregulated in tumors associated with high-volume ascites as compared to tumors associated with low-volume ascites

## Figures and Tables

**Table 1:** Gene ontology (GO) categories enriched in the low-volume ascites group.

GO term	GO accession	*P* value	Corrected *P* value
Antigen processing and presentation	GO:0019882/GO:0030333	7.56*E* − 10	1.45*E* − 06
Antigen processing and presentation of endogenous antigen	GO:0019883	6.37*E* − 04	0.1561
Antigen processing and presentation of peptide or polysaccharide antigen via MHC class II	GO:0002504	3.93*E* − 10	9.39*E* − 07
Chemokine activity	GO:0008009	3.80*E* − 06	0.0032
Chemokine receptor binding	GO:0042379	5.63*E* − 06	0.0041
Chemotaxis	GO:0006935	7.94*E* − 05	0.0362
Cytokine activity	GO:0005125	9.53*E* − 07	0.0010
Cytokine receptor binding	GO:0005126	1.44*E* − 04	0.0549
Defense response	GO:0006952/GO:0002217/GO:0042829	1.71*E* − 07	2.33*E* − 04
Extracellular region	GO:0005576	7.56*E* − 04	0.1764
Extracellular region part	GO:0044421	7.32*E* − 05	0.0362
Extracellular space	GO:0005615	4.03*E* − 06	0.0032
Fatty acid biosynthetic process	GO:0006633/GO:0000037	2.60*E* − 04	0.0890
G-protein-coupled receptor binding	GO:0001664	2.08*E* − 06	0.0020
Icosanoid biosynthetic process	GO:0046456	2.94*E* − 04	0.0971
Immune response	GO:0006955	1.69*E* − 13	8.08*E* − 10
Immune system process	GO:0002376	5.64*E* − 14	5.39*E* − 10
Inflammatory response	GO:0006954	2.28*E* − 05	0.0129
Locomotion	GO:0040011	6.67*E* − 04	0.1595
Membrane	GO:0016020	2.60*E* − 04	0.0890
Membrane raft	GO:0045121	1.34*E* − 04	0.0535
MHC class II protein complex	GO:0042613	8.24*E* − 11	2.63*E* − 07
MHC class II receptor activity	GO:0032395	3.53*E* − 07	4.22*E* − 04
MHC protein complex	GO:0042611	3.07*E* − 08	4.90*E* − 05
Multiorganism process	GO:0051704/GO:0051706	1.59*E* − 04	0.0583
Oligopeptide transport	GO:0006857	6.37*E* − 04	0.1561
Oligopeptide transporter activity	GO:0015198	6.37*E* − 04	0.1561
Phospholipid efflux	GO:0033700	8.16*E* − 04	0.1858
Polyamine biosynthetic process	GO:0006596	4.79*E* − 04	0.1273
Protein binding	GO:0005515/GO:0045308	3.67*E* − 04	0.1065
Receptor binding	GO:0005102	1.57*E* − 05	0.0094
Response to biotic stimulus	GO:0009607	5.09*E* − 05	0.0271
Response to other organisms	GO:0051707/GO:0009613/GO:0042828	5.97*E* − 06	0.0041
Response to stimulus	GO:0050896/GO:0051869	1.40*E* − 05	0.0089
Response to stress	GO:0006950	9.96*E* − 05	0.0414
Response to virus	GO:0009615	8.76*E* − 05	0.0381
Response to wounding	GO:0009611/GO:0002245	3.49*E* − 04	0.1065
Taxis	GO:0042330	7.94*E* − 05	0.0362
Unsaturated fatty acid biosynthetic process	GO:0006636	4.04*E* − 04	0.1138
Vacuolar membrane	GO:0005774	3.67*E* − 04	0.1065
Vacuolar part	GO:0044437	4.31*E* − 04	0.1179
Vacuole	GO:0005773	3.66*E* − 04	0.1065

**Table 2:** Immunostaining score for tumor infiltrating immune cells.

Variable	High-volume ascites N = 25	Low-volume ascites *N* = 26	*P* value*
CD20			
Median (IQR)	0 (0-0)	0 (0-1)	0.02
[Range]	[0–2]	[0–>50]	

CD8			
Median (IQR)	5 (0–11)	15 (10–29)	0.001
[Range]	[0–>50]	[2–>50]	

CD3			
Median (IQR)	5 (0–26)	15 (8–44)	0.01
[Range]	[0–>50]	[1–>50]	

*P values determined by Wilcoxon rank-sum test. IQR: interquartile range.

## Abbreviations

CTL: Cytotoxic T lymphocytes
EOC: Epithelial ovarian cancer
FDR: False discovery rate
FWER: Family wise error rate
GO: Gene ontology
GSEA: Gene set enrichment analysis
HGSOC: High-grade serous ovarian cancer
IQR: Interquartile range
TCGA: The cancer genome atlas
TILS: Tumor infiltrating lymphocytes
VEGF: Vascular endothelial growth factor.
