# Supplementary material for: Molecular Profiling and Clinical Outcome of High-Grade Serous Ovarian Cancer Presenting with Low- versus High-Volume Ascites
Source: Biomed Res Int. 2014 May 25;2014:367103. doi: 10.1155/2014/367103 (PMC4055662; doi:10.1155/2014/367103)
Supplement: Supplementary file 1 — Three supplemental tables are available online. The first of these provides a complete listing of the genes found differentially expressed between HGSOC tumors associated with low-volume ascites and tumors associated with high-volume ascites. The second table presents results of an independent gene set enrichment analysis (GSEA) of the gene expression data, which further supports the interpretation that genes up-regulated in tumors associated with low-volume ascites are involved in immune response. The third Table presents the results of a gene ontology (GO) analysis of genes upregulated in tumors associated with high-volume ascites as compared to tumors associated with low-volume ascites [file 367103.f1.pdf]

Supplemental Table S1: Probes differentially expressed in primary tumors from patients with low- and high-volume ascites as determined by a moderated t-test and with a minimum 1.5-fold cut off

*Regulation refers to low-volume ascites relative to high-volume ascites
Red highlights are Probes also significant with Westfall and Young FWER correction

Probe_Id	Symbol	Entrez_Gene_ID	Definition
ILMN_2393765	IGLL1	3543	Homo sapiens immunoglobulin lambda-like
ILMN_1695311	HLA-DMA	3108	Homo sapiens major histocompatibility com
ILMN_2376205	LTB	4050	Homo sapiens lymphotoxin beta (TNF supe
ILMN_1761733	HLA-DMB	3109	Homo sapiens major histocompatibility com
ILMN_3228688	LOC730415	730415	PREDICTED: Homo sapiens hypothetical L
ILMN_1791759	CXCL10	3627	Homo sapiens chemokine (C-X-C motif) liga
ILMN_2098126	CCL5	6352	Homo sapiens chemokine (C-C motif) ligan
ILMN_2157441	HLA-DRA	3122	Homo sapiens major histocompatibility com
ILMN_1798177	CHURC1	91612	Homo sapiens churchill domain containing
ILMN_1772218	HLA-DPA1	3113	Homo sapiens major histocompatibility com
ILMN_1752592	HLA-DRB4	3126	Homo sapiens major histocompatibility com
ILMN_2066066	HLA-DRB6	3128	Homo sapiens major histocompatibility com
ILMN_2083066	IGLL3	91353	Homo sapiens immunoglobulin lambda-like
ILMN_3241554	KANK4	163782	Homo sapiens KN motif and ankyrin repeat
ILMN_1751079	TAP1	6890	Homo sapiens transporter 1, ATP-binding c
ILMN_1662358	MX1	4599	Homo sapiens myxovirus (influenza virus) r
ILMN_1696657	LRRN2	10446	Homo sapiens leucine rich repeat neuronal
ILMN_1796316	MMP9	4318	Homo sapiens matrix metallopeptidase 9 (g
ILMN_2379644	CD74	972	Homo sapiens CD74 molecule, major histo
ILMN_1689655	HLA-DRA	3122	Homo sapiens major histocompatibility com
ILMN_2109708	ECGF1	1890	Homo sapiens endothelial cell growth facto
ILMN_1736567	CD74	972	Homo sapiens CD74 molecule, major histo
ILMN_1789007	APOC1	341	Homo sapiens apolipoprotein C-I (APOC1),
ILMN_1691364	STAT1	6772	Homo sapiens signal transducer and activa
ILMN_2368530	IL32	9235	Homo sapiens interleukin 32 (IL32), transcr
ILMN_2396444	CD14	929	Homo sapiens CD14 molecule (CD14), tran
ILMN_1815895	LOC649143	649143	PREDICTED: Homo sapiens similar to HLA
ILMN_2175912	ITGB2	3689	Homo sapiens integrin, beta 2 (complemen
ILMN_1714861	CD68	968	Homo sapiens CD68 antigen (CD68), mRN
ILMN_1729749	HERC5	51191	Homo sapiens hect domain and RLD 5 (HE
ILMN_1654396	ITGB2	3689	Homo sapiens integrin, beta 2 (antigen CD
ILMN_1740938	APOE	348	Homo sapiens apolipoprotein E (APOE), m
ILMN_2103107	ADAMDEC1	27299	Homo sapiens ADAM-like, decysin 1 (ADAM
ILMN_1745356	CXCL9	4283	Homo sapiens chemokine (C-X-C motif) liga
ILMN_3243714	LOC642073	642073	PREDICTED: Homo sapiens similar to MH
ILMN_1728478	CXCL16	58191	Homo sapiens chemokine (C-X-C motif) liga
ILMN_1674063	OAS2	4939	Homo sapiens 2'-5'-oligoadenylate syntheta
ILMN_2246083	C7orf28B	221960	Homo sapiens chromosome 7 open reading
ILMN_3249667	LOC100133678	100133678	PREDICTED: Homo sapiens similar to hCG
ILMN_1808405	HLA-DQA1	3117	PREDICTED: Homo sapiens major histoco
ILMN_1690105	STAT1	6772	Homo sapiens signal transducer and activa
ILMN_1804663	THBS3	7059	Homo sapiens thrombospondin 3 (THBS3),
ILMN_2388547	EPSTI1	94240	Homo sapiens epithelial stromal interaction
ILMN_1669928	ARHGEF16	27237	Homo sapiens Rho guanine exchange facto

ILMN_1733811	JUP	3728	Homo sapiens junction plakoglobin (JUP), t
ILMN_1812031	PALM	5064	Homo sapiens paralemmin (PALM), transcr
ILMN_2075189	SLC35F2	54733	Homo sapiens solute carrier family 35, mem
ILMN_1772964	CCL8	6355	Homo sapiens chemokine (C-C motif) ligan
ILMN_1682864	SPSB3	90864	Homo sapiens splA/ryanodine receptor dom
ILMN_1701413	PIGQ	9091	Homo sapiens phosphatidylinositol glycan a
ILMN_2387471	FLJ22184	80164	Homo sapiens hypothetical protein FLJ2218
ILMN_1796409	C1QB	713	Homo sapiens complement component 1, q
ILMN_1727479	TPRG1L	127262	Homo sapiens tumor protein p63 regulated
ILMN_1665909	LASP1	3927	Homo sapiens LIM and SH3 protein 1 (LAS
ILMN_2115218	ANKRD10	55608	Homo sapiens ankyrin repeat domain 10 (A
ILMN_1723042	CLDN3	1365	Homo sapiens claudin 3 (CLDN3), mRNA.
ILMN_1781373	IFIH1	64135	Homo sapiens interferon induced with helic
ILMN_3233930	LOC390557	390557	PREDICTED: Homo sapiens hypothetical L
ILMN_2352121	NT5C3	51251	Homo sapiens 5'-nucleotidase, cytosolic III
ILMN_1680996	ALOX5	240	PREDICTED: Homo sapiens arachidonate
ILMN_1800451	MED16	10025	Homo sapiens mediator complex subunit 16
ILMN_1693826	HAVCR2	84868	Homo sapiens hepatitis A virus cellular rece
ILMN_1715607	CHMP4A	29082	Homo sapiens chromatin modifying protein
ILMN_1730612	DBNDD2	55861	Homo sapiens dysbindin (dystrobrevin bind
ILMN_1694731	CLCN7	1186	Homo sapiens chloride channel 7 (CLCN7),
ILMN_1729237	CYB5R1	51706	Homo sapiens cytochrome b5 reductase 1 (
ILMN_2114568	GBP5	115362	Homo sapiens guanylate binding protein 5 (
ILMN_1752579	ATP6V0A1	535	Homo sapiens ATPase, H+ transporting, lys
ILMN_1678671	KLHL24	54800	Homo sapiens kelch-like 24 (Drosophila) (K
ILMN_1809477	CARHSP1	23589	Homo sapiens calcium regulated heat stabl
ILMN_1807206	DHRS1	115817	Homo sapiens dehydrogenase/reductase (S
ILMN_2327994	AZIN1	51582	Homo sapiens antizyme inhibitor 1 (AZIN1)
ILMN_2337655	WARS	7453	Homo sapiens tryptophanyl-tRNA synthetas
ILMN_2074860	RN7SK	125050	Homo sapiens RNA, 7SK small nuclear (RN
ILMN_1811702	GRN	2896	Homo sapiens granulin (GRN), mRNA.
ILMN_1780825	RRAS	6237	Homo sapiens related RAS viral (r-ras) onc
ILMN_1694479	WDR18	57418	Homo sapiens WD repeat domain 18 (WDR
ILMN_1772731	HAGH	3029	Homo sapiens hydroxyacylglutathione hydr
ILMN_1688642	LAMC3	10319	Homo sapiens laminin, gamma 3 (LAMC3),
ILMN_1801491	SLC26A2	1836	Homo sapiens solute carrier family 26 (sulfa
ILMN_1659610	TJP3	27134	Homo sapiens tight junction protein 3 (zona
ILMN_2332105	WRNIP1	56897	Homo sapiens Werner helicase interacting
ILMN_2104106	XPR1	9213	Homo sapiens xenotropic and polytropic ret
ILMN_1669966	NDUFS7	374291	Homo sapiens NADH dehydrogenase (ubiq
ILMN_1669878	GUSB	2990	Homo sapiens glucuronidase, beta (GUSB)
ILMN_2162972	LYZ	4069	Homo sapiens lysozyme (renal amyloidosis
ILMN_1691731	PARP14	54625	Homo sapiens poly (ADP-ribose) polymeras
ILMN_1777565	TAP2	6891	Homo sapiens transporter 2, ATP-binding c
ILMN_2111187	ELOVL6	79071	Homo sapiens ELOVL family member 6, elo
ILMN_1738347	RNPEP	6051	Homo sapiens arginyl aminopeptidase (ami
ILMN_2383693	UPF2	26019	Homo sapiens UPF2 regulator of nonsense
ILMN_1789005	ATP6V0C	527	PREDICTED: Homo sapiens ATPase, H+ tr
ILMN_1698243	C1orf85	112770	Homo sapiens chromosome 1 open reading
ILMN_2060413	CD24	100133941	Homo sapiens CD24 molecule (CD24), mR
ILMN_2061043	CD48	962	Homo sapiens CD48 molecule (CD48), mR

ILMN_1682930	SIPA1	6494	Homo sapiens signal-induced proliferation-a
ILMN_1773080	OAZ1	4946	Homo sapiens ornithine decarboxylase anti
ILMN_1699931	HCST	10870	Homo sapiens hematopoietic cell signal tra
ILMN_1712705	RAB40C	57799	Homo sapiens RAB40C, member RAS onco
ILMN_2115005	FGD2	221472	Homo sapiens FYVE, RhoGEF and PH don
ILMN_1769734	NT5C3	51251	Homo sapiens 5'-nucleotidase, cytosolic III
ILMN_1764764	MUM1	84939	Homo sapiens melanoma associated antige
ILMN_1763000	ADAP2	55803	Homo sapiens ArfGAP with dual PH domai
ILMN_1667796	HBA2	3040	Homo sapiens hemoglobin, alpha 2 (HBA2)
ILMN_3297947	LOC728368	728368	PREDICTED: Homo sapiens misc_RNA (LO
ILMN_2319424	GYG2	8908	Homo sapiens glycogenin 2 (GYG2), transc
ILMN_1827736			Homo sapiens cDNA clone IMAGE:526273
ILMN_2380698	DSTN	11034	Homo sapiens destrin (actin depolymerizing
ILMN_1651557	KDELC2	143888	Homo sapiens KDEL (Lys-Asp-Glu-Leu) co
ILMN_2247594	RPLP1	6176	Homo sapiens ribosomal protein, large, P1
ILMN_3177691	LOC100130750	100130750	PREDICTED: Homo sapiens misc_RNA (LO
ILMN_1770053	RBBP7	5931	Homo sapiens retinoblastoma binding prote
ILMN_1714082	CMAS	55907	Homo sapiens cytidine monophosphate N-a
ILMN_3201216	LOC441550	441550	PREDICTED: Homo sapiens misc_RNA (LO
ILMN_1795243	LOC220433	220433	PREDICTED: Homo sapiens similar to 40S
ILMN_1683678	SPATS2L	26010	Homo sapiens spermatogenesis associated
ILMN_1800659	PGM1	5236	Homo sapiens phosphoglucomutase 1 (PG
ILMN_2051684	LOC401152	401152	Homo sapiens HCV F-transactivated protei
ILMN_3283772	LOC644237	644237	PREDICTED: Homo sapiens misc_RNA (LO
ILMN_1343295	GAPDH	2597	Homo sapiens glyceraldehyde-3-phosphate
ILMN_1689088	COLEC12	81035	Homo sapiens collectin sub-family member
ILMN_1727134	KLHDC5	57542	Homo sapiens kelch domain containing 5 (K
ILMN_3205162	LOC646688	646688	PREDICTED: Homo sapiens misc_RNA (LO
ILMN_1684554	COL16A1	1307	Homo sapiens collagen, type XVI, alpha 1 (
ILMN_2308849	MYADM	91663	Homo sapiens myeloid-associated different
ILMN_1713394	LOC441876	441876	PREDICTED: Homo sapiens similar to 40S
ILMN_1802252	GAPDH	2597	Homo sapiens glyceraldehyde-3-phosphate
ILMN_2130180	RPL13L	283345	Homo sapiens ribosomal protein L13-like (R
ILMN_1762899	EGR1	1958	Homo sapiens early growth response 1 (EG
ILMN_1810577	RPS4X	6191	Homo sapiens ribosomal protein S4, X-linke
ILMN_2360415	PRNP	5621	Homo sapiens prion protein (PRNP), transc
ILMN_2207533	RPS17	6218	Homo sapiens ribosomal protein S17 (RPS
ILMN_2350634	EFEMP1	2202	Homo sapiens EGF-containing fibulin-like e
ILMN_1733756	COL12A1	1303	Homo sapiens collagen, type XII, alpha 1 (C
ILMN_1746359	RERG	85004	Homo sapiens RAS-like, estrogen-regulated
ILMN_1728011	NLGN4X	57502	Homo sapiens neuroligin 4, X-linked (NLGN
ILMN_2375879	VEGFA	7422	Homo sapiens vascular endothelial growth
ILMN_2059689	TMEM54	113452	Homo sapiens transmembrane protein 54 (T
ILMN_1758164	STC1	6781	Homo sapiens stanniocalcin 1 (STC1), mRN
ILMN_2038778	GAPDH	2597	Homo sapiens glyceraldehyde-3-phosphate
ILMN_1707810	RPS5	6193	Homo sapiens ribosomal protein S5 (RPS5)
ILMN_1689725	RPLP1	6176	Homo sapiens ribosomal protein, large, P1
ILMN_1751097	CREB3L2	64764	Homo sapiens cAMP responsive element b
ILMN_1681780	MKX	283078	Homo sapiens mohawk homeobox (MKX), r
ILMN_3205656	LOC391075	391075	PREDICTED: Homo sapiens misc_RNA (LO
ILMN_1777286	LOC388344	388344	PREDICTED: Homo sapiens similar to ribos

ILMN_1664610	LOC402057	402057	Homo sapiens similar to 40S ribosomal pro
ILMN_1653203	EFEMP2	30008	Homo sapiens EGF-containing fibulin-like e
ILMN_2125374	CMAS	55907	Homo sapiens cytidine monophosphate N-a
ILMN_2129234	TMEM47	83604	Homo sapiens transmembrane protein 47 (
ILMN_1662640	C20orf127	140851	Homo sapiens chromosome 20 open readir
ILMN_2395451	ASS1	445	Homo sapiens argininosuccinate synthetas
ILMN_2386530	RPLP1	6176	Homo sapiens ribosomal protein, large, P1
ILMN_1784706	GABRE	2564	Homo sapiens gamma-aminobutyric acid (G
ILMN_1712305	CYBRD1	79901	Homo sapiens cytochrome b reductase 1 (C
ILMN_3199929	LOC390183	390183	PREDICTED: Homo sapiens misc_RNA (LO
ILMN_1768110	ZAK	51776	Homo sapiens sterile alpha motif and leucir
ILMN_1684873	ARSD	414	Homo sapiens arylsulfatase D (ARSD), tran
ILMN_1659027	SLC2A1	6513	Homo sapiens solute carrier family 2 (facilit
ILMN_1708778	ASS1	445	Homo sapiens argininosuccinate synthetas
ILMN_2166831	RPS4X	6191	Homo sapiens ribosomal protein S4, X-linke
ILMN_1793476	PRKCDBP	112464	Homo sapiens protein kinase C, delta bindi
ILMN_1707627	TPI1	7167	Homo sapiens triosephosphate isomerase 1
ILMN_1676088	MSRB3	253827	Homo sapiens methionine sulfoxide reducta
ILMN_1691156	MT1A	4489	Homo sapiens metallothionein 1A (MT1A),
ILMN_1709634	CMBL	134147	Homo sapiens carboxymethylenebutenolida
ILMN_2087692	CYBRD1	79901	Homo sapiens cytochrome b reductase 1 (C
ILMN_1733415	MFAP5	8076	Homo sapiens microfibrillar associated prot
ILMN_1672503	DPYSL2	1808	Homo sapiens dihydropyrimidinase-like 2 (D
ILMN_1796734	SPARC	6678	Homo sapiens secreted protein, acidic, cyst
ILMN_1704537	PHGDH	26227	Homo sapiens phosphoglycerate dehydrog
ILMN_1802205	RHOB	388	Homo sapiens ras homolog gene family, me
ILMN_1732151	COL6A1	1291	Homo sapiens collagen, type VI, alpha 1 (C
ILMN_1806023	JUN	3725	Homo sapiens jun oncogene (JUN), mRNA
ILMN_1796712	S100A10	6281	Homo sapiens S100 calcium binding protei
ILMN_2046730	S100A10	6281	Homo sapiens S100 calcium binding protei
ILMN_1776188	MAP1LC3A	84557	Homo sapiens microtubule-associated prote
ILMN_1775708	SLC2A3	6515	Homo sapiens solute carrier family 2 (facilit
ILMN_3305475	LOC729708	729708	PREDICTED: Homo sapiens similar to rcTP
ILMN_1729453	TSPAN9	10867	Homo sapiens tetraspanin 9 (TSPAN9), mR
ILMN_1737988	PRNP	5621	Homo sapiens prion protein (PRNP), transc
ILMN_1713561	C20orf103	24141	Homo sapiens chromosome 20 open readir
ILMN_1730487	CALD1	800	Homo sapiens caldesmon 1 (CALD1), trans
ILMN_1792455	TMEM158	25907	Homo sapiens transmembrane protein 158
ILMN_2100437	HBB	3043	Homo sapiens hemoglobin, beta (HBB), mR
ILMN_1672536	FBLN1	2192	Homo sapiens fibulin 1 (FBLN1), transcript
ILMN_2341067	NLGN4X	57502	Homo sapiens neuroligin 4, X-linked (NLGN
ILMN_1754795	FAT1	2195	Homo sapiens FAT tumor suppressor homo
ILMN_2149226	CAV1	857	Homo sapiens caveolin 1, caveolae protein
ILMN_1708934	ADM	133	Homo sapiens adrenomedullin (ADM), mRN
ILMN_2173611	MT1E	4493	Homo sapiens metallothionein 1E (MT1E),
ILMN_2188264	CYR61	3491	Homo sapiens cysteine-rich, angiogenic inc
ILMN_1661599	DDIT4	54541	Homo sapiens DNA-damage-inducible trans
ILMN_1718766	MT1F	4494	Homo sapiens metallothionein 1F (MT1F), r
ILMN_1715401	MT1G	4495	Homo sapiens metallothionein 1G (MT1G),
ILMN_1781285	DUSP1	1843	Homo sapiens dual specificity phosphatase
ILMN_1751607	FOSB	2354	Homo sapiens FBJ murine osteosarcoma v

ILMN_1669523	FOS	2353	Homo sapiens v-fos FBJ murine osteosarc

p (Corr)	p	Regulation*	FC (abs)	FC	Log FC
1	0.007691435	UP	4.799252	-4.799252	-2.2628095
0.28282827	0.001943686	UP	3.1758215	-3.1758215	-1.6671299
1	0.010950411	UP	2.9188452	-2.9188452	-1.5453976
0.01010101	2.65E-004	UP	2.8268652	-2.8268652	-1.4992031
0.83838385	0.003274125	UP	2.8061256	-2.8061256	-1.4885796
1	0.014933238	UP	2.7765532	-2.7765532	-1.473295
0.05050505	9.85E-004	UP	2.7103949	-2.7103949	-1.438503
1	0.01673172	UP	2.619494	-2.619494	-1.3892881
0.121212125	0.001445771	UP	2.588296	-2.588296	-1.3720026
0.02020202	6.48E-004	UP	2.5692863	-2.5692863	-1.3613677
0.030303031	7.87E-004	UP	2.4985914	-2.4985914	-1.321115
0.94949496	0.004227277	UP	2.4940672	-2.4940672	-1.3185003
1	0.017644422	UP	2.452279	-2.452279	-1.2941232
1	0.044879768	UP	2.3595896	-2.3595896	-1.2385359
1	0.022124395	UP	2.3539748	-2.3539748	-1.2350988
1	0.023775402	UP	2.3314228	-2.3314228	-1.2212106
1	0.01754079	UP	2.32864	-2.32864	-1.2194875
0.5959596	0.002527964	UP	2.3008506	-2.3008506	-1.2021673
1	0.02529319	UP	2.2260497	-2.2260497	-1.1544858
1	0.009291469	UP	2.2025473	-2.2025473	-1.139173
1	0.04274278	UP	2.1970098	-2.1970098	-1.1355413
1	0.029788107	UP	2.1162233	-2.1162233	-1.0814918
1	0.010018742	UP	2.0851407	-2.0851407	-1.0601448
1	0.011990373	UP	2.0324802	-2.0324802	-1.0232413
1	0.009013817	UP	2.018056	-2.018056	-1.0129662
1	0.04169217	UP	2.0040784	-2.0040784	-1.002939
1	0.01745142	UP	1.9863731	-1.9863731	-0.99013656
1	0.037032034	UP	1.9819747	-1.9819747	-0.98693854
1	0.015061763	UP	1.9780812	-1.9780812	-0.98410165
1	0.014807519	UP	1.9551288	-1.9551288	-0.96726364
1	0.04362536	UP	1.9541819	-1.9541819	-0.9665648
1	0.023217257	UP	1.9289486	-1.9289486	-0.9478147
0.05050505	0.001067722	UP	1.9220462	-1.9220462	-0.942643
1	0.03294853	UP	1.8772472	-1.8772472	-0.9086186
1	0.005137109	UP	1.8649485	-1.8649485	-0.89913577
1	0.00496161	UP	1.8110956	-1.8110956	-0.85686266
1	0.039143156	UP	1.7985386	-1.7985386	-0.84682506
1	0.026397184	UP	1.7972615	-1.7972615	-0.8458003
0.969697	0.004341777	UP	1.797151	-1.797151	-0.8457116
0.85858583	0.003574868	UP	1.7831486	-1.7831486	-0.83442694
1	0.006234104	UP	1.765034	-1.765034	-0.81969595
1	0.008768678	UP	1.7464391	-1.7464391	-0.8044163
1	0.024091545	UP	1.7461265	-1.7461265	-0.8041581
0.11111111	0.001329151	UP	1.7174714	-1.7174714	-0.780286

0.25252524	0.001880808	UP	1.7066371	-1.7066371	-0.7711564
0.7777778	0.003006373	UP	1.7004381	-1.7004381	-0.7659065
1	0.043190513	UP	1.6850723	-1.6850723	-0.7528105
1	0.006613552	UP	1.6813649	-1.6813649	-0.74963284
0.82828283	0.003231082	UP	1.677676	-1.677676	-0.7464641
0.17171717	0.001642084	UP	1.6758196	-1.6758196	-0.74486685
1	0.016931146	UP	1.6738061	-1.6738061	-0.74313235
1	0.0418709	UP	1.6691117	-1.6691117	-0.73908055
1	0.02257376	UP	1.6637845	-1.6637845	-0.7344686
0.17171717	0.001615166	UP	1.6629092	-1.6629092	-0.73370934
0.4848485	0.002372029	UP	1.6626135	-1.6626135	-0.73345286
1	0.015424254	UP	1.6612636	-1.6612636	-0.73228097
1	0.04315604	UP	1.6526841	-1.6526841	-0.72481096
1	0.022914968	UP	1.6493729	-1.6493729	-0.7219176
1	0.009550892	UP	1.6475449	-1.6475449	-0.7203177
1	0.032069523	UP	1.6467375	-1.6467375	-0.7196106
0.09090909	0.001289698	UP	1.6312653	-1.6312653	-0.7059914
1	0.02867055	UP	1.6291533	-1.6291533	-0.7041223
1	0.008994055	UP	1.629013	-1.629013	-0.703998
1	0.041174084	UP	1.6288667	-1.6288667	-0.7038685
1	0.00518489	UP	1.619323	-1.619323	-0.69539076
1	0.012629087	UP	1.6049693	-1.6049693	-0.68254566
0.15151516	0.001537626	UP	1.6012185	-1.6012185	-0.67917013
0.15151516	0.001540735	UP	1.5989845	-1.5989845	-0.6771559
1	0.008046195	UP	1.598463	-1.598463	-0.6766854
1	0.029454762	UP	1.5950463	-1.5950463	-0.6735983
1	0.013489665	UP	1.5940878	-1.5940878	-0.6727311
1	0.00820983	UP	1.5847394	-1.5847394	-0.66424567
1	0.018856972	UP	1.5810095	-1.5810095	-0.66084605
1	0.034690663	UP	1.5789642	-1.5789642	-0.65897846
0.72727275	0.002849182	UP	1.5784736	-1.5784736	-0.6585301
1	0.038808204	UP	1.5739187	-1.5739187	-0.654361
0.7777778	0.003080587	UP	1.57188	-1.57188	-0.65249103
1	0.018907834	UP	1.5702616	-1.5702616	-0.6510049
1	0.044984758	UP	1.5662098	-1.5662098	-0.6472775
1	0.015438386	UP	1.5611229	-1.5611229	-0.6425841
1	0.02445228	UP	1.5602282	-1.5602282	-0.6417571
1	0.010293297	UP	1.5592669	-1.5592669	-0.6408679
1	0.008788483	UP	1.5580956	-1.5580956	-0.63978374
1	0.009309723	UP	1.557785	-1.557785	-0.63949615
1	0.005378605	UP	1.5573534	-1.5573534	-0.6390963
1	0.005837087	UP	1.55045	-1.55045	-0.632687
1	0.022409001	UP	1.5470349	-1.5470349	-0.6295057
0.11111111	0.001339337	UP	1.5459976	-1.5459976	-0.6285381
1	0.03264293	UP	1.5381083	-1.5381083	-0.6211571
1	0.006159702	UP	1.5376188	-1.5376188	-0.62069786
0.969697	0.004427246	UP	1.5326377	-1.5326377	-0.6160167
1	0.005704618	UP	1.529167	-1.529167	-0.612746
1	0.008046531	UP	1.5266833	-1.5266833	-0.61040086
1	0.020432154	UP	1.526098	-1.526098	-0.6098476
0.030303031	6.75E-004	UP	1.5228895	-1.5228895	-0.6068112

1	0.007774072	UP	1.5225998	-1.5225998	-0.6065368
0	5.60E-005	UP	1.5210675	-1.5210675	-0.6050842
1	0.015353091	UP	1.5198573	-1.5198573	-0.60393584
0.01010101	3.22E-004	UP	1.5162688	-1.5162688	-0.60052556
1	0.04656766	UP	1.5155698	-1.5155698	-0.5998603
1	0.006251735	UP	1.506527	-1.506527	-0.59122646
1	0.012454917	UP	1.5062647	-1.5062647	-0.5909753
1	0.014670901	UP	1.5018586	-1.5018586	-0.58674896
1	0.026521664	DOWN	1.5024333	1.5024333	0.58730096
0.94949496	0.00421235	DOWN	1.5033958	1.5033958	0.5882249
1	0.041957963	DOWN	1.5063382	1.5063382	0.59104574
1	0.035786964	DOWN	1.5101643	1.5101643	0.59470546
1	0.046056345	DOWN	1.5129976	1.5129976	0.5974097
1	0.04047794	DOWN	1.5187292	1.5187292	0.6028646
1	0.013641273	DOWN	1.5187676	1.5187676	0.6029011
1	0.006338574	DOWN	1.5205492	1.5205492	0.60459244
1	0.034224134	DOWN	1.5281091	1.5281091	0.6117475
1	0.004945448	DOWN	1.5303737	1.5303737	0.613884
1	0.016218321	DOWN	1.5322486	1.5322486	0.6156504
1	0.015699606	DOWN	1.5375173	1.5375173	0.62060267
1	0.017509945	DOWN	1.5431328	1.5431328	0.6258622
1	0.014940954	DOWN	1.5489773	1.5489773	0.63131595
1	0.017919736	DOWN	1.5491439	1.5491439	0.63147116
0.33333334	0.002017794	DOWN	1.5532614	1.5532614	0.63530064
0.08080808	0.001247029	DOWN	1.5537333	1.5537333	0.6357389
1	0.049487554	DOWN	1.5541135	1.5541135	0.6360919
1	0.015164889	DOWN	1.5586059	1.5586059	0.64025617
0.989899	0.00475682	DOWN	1.5643737	1.5643737	0.6455852
1	0.038844056	DOWN	1.5657543	1.5657543	0.6468578
1	0.04673143	DOWN	1.5672348	1.5672348	0.6482213
1	0.01088111	DOWN	1.5707736	1.5707736	0.65147525
0.83838385	0.003315591	DOWN	1.5721841	1.5721841	0.65277016
0.9292929	0.004080203	DOWN	1.5735493	1.5735493	0.65402234
1	0.018315952	DOWN	1.5758042	1.5758042	0.6560883
1	0.010845139	DOWN	1.5817118	1.5817118	0.66148674
1	0.012819028	DOWN	1.5861524	1.5861524	0.6655314
0.969697	0.004347692	DOWN	1.5868943	1.5868943	0.666206
1	0.043302137	DOWN	1.5887548	1.5887548	0.66789645
1	0.006801711	DOWN	1.589618	1.589618	0.6686801
0.9292929	0.004054786	DOWN	1.5915092	1.5915092	0.6703955
1	0.022889167	DOWN	1.5996246	1.5996246	0.6777334
1	0.0488545	DOWN	1.600928	1.600928	0.67890835
1	0.030164508	DOWN	1.6047238	1.6047238	0.682325
1	0.024440683	DOWN	1.6115845	1.6115845	0.68847984
0.18181819	0.00166122	DOWN	1.6135684	1.6135684	0.69025475
1	0.011548934	DOWN	1.6138971	1.6138971	0.6905486
1	0.027061561	DOWN	1.616362	1.616362	0.69275033
1	0.034832872	DOWN	1.6269795	1.6269795	0.70219606
1	0.02536943	DOWN	1.6285919	1.6285919	0.70362514
0.060606062	0.001152991	DOWN	1.6301616	1.6301616	0.705015
1	0.015315795	DOWN	1.6394435	1.6394435	0.7132062

0.3131313	0.002005578	DOWN	1.6508459	1.6508459	0.72320545
1	0.03667991	DOWN	1.6524019	1.6524019	0.7245646
1	0.006158799	DOWN	1.6537937	1.6537937	0.72577924
1	0.03562813	DOWN	1.6541766	1.6541766	0.72611326
1	0.014653291	DOWN	1.6865356	1.6865356	0.7540628
1	0.034009233	DOWN	1.6921077	1.6921077	0.75882137
1	0.011208121	DOWN	1.6967322	1.6967322	0.76275885
1	0.010930384	DOWN	1.6967889	1.6967889	0.7628071
1	0.031841163	DOWN	1.6980779	1.6980779	0.76390266
1	0.010106704	DOWN	1.710241	1.710241	0.7741996
1	0.035454705	DOWN	1.71103	1.71103	0.77486503
1	0.004830824	DOWN	1.7139913	1.7139913	0.7773598
1	0.03663659	DOWN	1.7153968	1.7153968	0.7785423
1	0.031968873	DOWN	1.7248499	1.7248499	0.78647083
1	0.005853472	DOWN	1.730538	1.730538	0.7912206
1	0.008402999	DOWN	1.7449756	1.7449756	0.8032068
1	0.014029294	DOWN	1.7580591	1.7580591	0.8139836
1	0.007208883	DOWN	1.7623824	1.7623824	0.817527
1	0.030202853	DOWN	1.7625744	1.7625744	0.8176842
1	0.04687567	DOWN	1.7627488	1.7627488	0.8178269
1	0.041963972	DOWN	1.7638112	1.7638112	0.81869614
1	0.025688829	DOWN	1.8199583	1.8199583	0.86390543
1	0.04226047	DOWN	1.8214407	1.8214407	0.86508
1	0.031217001	DOWN	1.8215812	1.8215812	0.86519134
1	0.040813293	DOWN	1.8400285	1.8400285	0.87972814
1	0.03919907	DOWN	1.8732387	1.8732387	0.90553474
1	0.007202414	DOWN	1.8766801	1.8766801	0.90818274
1	0.016655732	DOWN	1.8794011	1.8794011	0.91027296
1	0.023617832	DOWN	1.8949435	1.8949435	0.9221548
1	0.022621391	DOWN	1.9003344	1.9003344	0.92625326
1	0.010146182	DOWN	1.9081507	1.9081507	0.9321751
1	0.04945883	DOWN	1.9196507	1.9196507	0.94084376
0.02020202	4.87E-004	DOWN	1.9344338	1.9344338	0.95191133
1	0.017906472	DOWN	1.9373955	1.9373955	0.95411843
1	0.021104697	DOWN	1.96315	1.96315	0.9731704
1	0.006607299	DOWN	1.9697472	1.9697472	0.9780105
0.68686867	0.002758033	DOWN	1.9768394	1.9768394	0.98319566
1	0.012913531	DOWN	2.0000072	2.0000072	1.0000051
1	0.030624501	DOWN	2.0079334	2.0079334	1.0057114
1	0.012794066	DOWN	2.0282693	2.0282693	1.0202492
1	0.02677563	DOWN	2.0871613	2.0871613	1.061542
0.97979796	0.004659777	DOWN	2.1299665	2.1299665	1.0908307
0.01010101	1.48E-004	DOWN	2.2187963	2.2187963	1.1497772
1	0.00847594	DOWN	2.2404315	2.2404315	1.1637766
1	0.015709817	DOWN	2.3071432	2.3071432	1.2061075
1	0.01784236	DOWN	2.3794377	2.3794377	1.2506206
1	0.007131056	DOWN	2.460769	2.460769	1.2991092
0.05050505	9.99E-004	DOWN	2.4829366	2.4829366	1.3120474
1	0.0333096	DOWN	2.576056	2.576056	1.3651639
1	0.013629663	DOWN	2.632814	2.632814	1.3966055
1	0.022279652	DOWN	2.7629485	2.7629485	1.4662087

0.01010101	2.05E-004	DOWN	4.224708	4.224708	2.0788517
